# Investigation of the Effect of Curcumin on Protein Targets in NAFLD Using Bioinformatic Analysis

**DOI:** 10.3390/nu14071331

**Published:** 2022-03-22

**Authors:** Ali Mahmoudi, Alexandra E. Butler, Muhammed Majeed, Maciej Banach, Amirhossein Sahebkar

**Affiliations:** 1 Department of Medical Biotechnology and Nanotechnology, Faculty of Medicine, Mashhad University of Medical Sciences, Mashhad 9177899191, Iran; alimahmoudi68@yahoo.com; 2 Research Department, Royal College of Surgeons in Ireland Bahrain, Adliya 15503, Bahrain; aeb91011@gmail.com; 3 Sabinsa Corporation, East Windsor, NJ 08520, USA; mmjd52@hotmail.com; 4 Nephrology and Hypertension, Department of Preventive Cardiology and Lipidology, Medical University of Lodz, 93-338 Lodz, Poland; 5 Cardiovascular Research Centre, University of Zielona Gora, 65-417 Zielona Gora, Poland; 6 Biotechnology Research Center, Pharmaceutical Technology Institute, Mashhad University of Medical Sciences, Mashhad 9177899191, Iran; 7 Applied Biomedical Research Center, Mashhad University of Medical Sciences, Mashhad 9177899191, Iran; 8 Department of Biotechnology, School of Pharmacy, Mashhad University of Medical Sciences, Mashhad 9177899191, Iran

**Keywords:** curcumin, cell, tissue, gene ontology, KEGG, NAFLD, STITCH

## Abstract

BACKGROUND: Non-alcoholic fatty liver disease (NAFLD) is a prevalent metabolic disorder. Defects in function/expression of genes/proteins are critical in initiation/progression of NAFLD. Natural products may modulate these genes/proteins. Curcumin improves steatosis, inflammation, and fibrosis progression. Here, bioinformatic tools, gene–drug and gene-disease databases were utilized to explore targets, interactions, and pathways through which curcumin could impact NAFLD. METHODS: Significant curcumin–protein interaction was identified (high-confidence:0.7) in the STITCH database. Identified proteins were investigated to determine association with NAFLD. gene ontology (GO) and Kyoto Encyclopedia of Genes and Genomes (KEGG) were analyzed for significantly involved targets (*p* < 0.01). Specificity of obtained targets with NAFLD was estimated and investigated in Tissue/Cells–gene associations (PanglaoDB Augmented 2021, Mouse Gene Atlas) and Disease–gene association-based EnrichR algorithms (Jensen DISEASES, DisGeNET). RESULTS: Two collections were constructed: 227 protein–curcumin interactions and 95 NAFLD-associated genes. By Venn diagram, 14 significant targets were identified, and their biological pathways evaluated. Based on gene ontology, most targets involved stress and lipid metabolism. KEGG revealed chemical carcinogenesis, the AGE-RAGE signaling pathway in diabetic complications and NAFLD as the most common significant pathways. Specificity to diseases database (EnrichR algorithm) revealed specificity for steatosis/steatohepatitis. CONCLUSION: Curcumin may improve, or inhibit, progression of NAFLD through activation/inhibition of NAFLD-related genes.

## 1. Introduction

In recent decades, non-alcoholic fatty liver disease (NAFLD) has attracted interest from researchers as a highly prevalent metabolic disorder. Metabolic dysfunction can be initiated when there is only 5% fat accumulation in the liver. Furthermore, while an increasing proportion of the population has been diagnosed with NAFLD, scientific studies suggest that many individuals are unaware of the presence of the disease, which complicates the therapy [1]. NAFLD comprises a spectrum of liver disorders, from hepatic steatosis to non-alcoholic steatohepatitis (NASH) and, if unchecked, may progress to fibrosis and cirrhosis [2]. NAFLD is recognized to be closely associated with other metabolic disorders such as hyperlipidemia, obesity, hypertension, cardiovascular disease, and insulin resistance [3,4]. The global prevalence of NAFLD is approximately ~25%, with the highest prevalence in the Middle East. The prevalence of NAFLD in Asia has been determined to be 52.3 per 1000 people years [5]. Despite the health burden it imposes, no specific drugs have been approved for the treatment of NAFLD, though various therapeutic approaches have been proposed. Lifestyle intervention and pharmacological interventions are the mainstays of treatment for patients with NAFLD [6]. A number of studies have identified defects in the function or expression of genes and proteins as critical factors in the initiation and progression of NAFLD [7,8]. Various natural and chemical drugs are being studied to determine whether they modulate these genes and proteins [9,10]. One of the natural products that has drawn much attention for the treatment of metabolic diseases is curcumin.

Curcumin is a bioactive polyphenolic compound, isolated from *Curcuma longa* Linn, which is endowed with diverse pharmacological activities [11,12,13,14,15,16,17,18,19,20,21,22]. Numerous in-vitro and in-vivo investigations have indicated that curcumin exerts a positive effect at each stage of NAFLD, improving both inflammation and the extent of fat deposition. Curcumin was also shown to inhibit the progression from NAFLD to fibrosis and decrease the risk of liver cancer. More recently, several clinical trials have reported beneficial changes, including a decrease in inflammation, increased antioxidant factors, and improvement in liver histopathology [23,24,25,26,27,28,29,30,31].

Emerging from these studies, therefore, are data supporting the significant clinical effect of curcumin on NAFLD. Although extensive data exist on the benefits of curcumin in NAFLD, studies on the mechanism of action of curcumin and its interaction with various genes have not been evaluated in detail.

Systems biology and bioinformatics, a branch of biology that combines molecular biology and computer science, have been an integral part of research investigations in recent decades. This science focuses on the entire biological process and system as compared to past years where there was a tendency to investigate only a single protein or gene [32].

**Figure 1:**
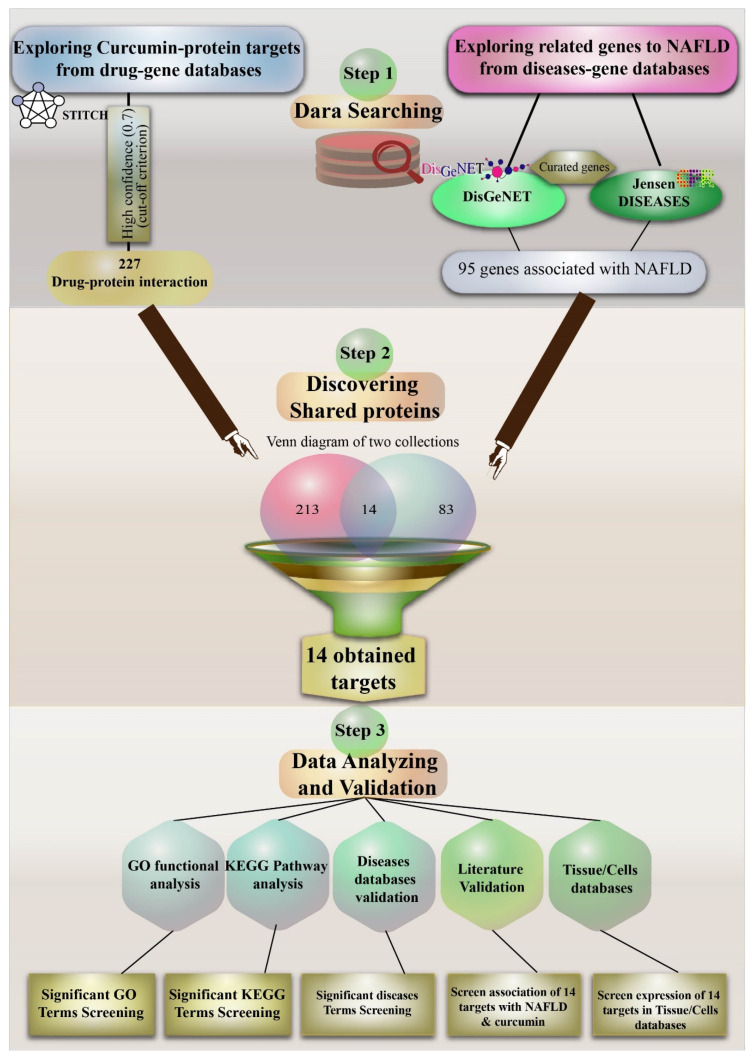
A comprehensive diagram illustrating the investigative strategy undertaken in the present study. Non-alcoholic fatty liver disease (NAFLD); gene ontology (GO); Kyoto Encyclopedia of Genes and Genomes (KEGG).

In this bioinformatics study, we aimed to identify important gene targets for NAFLD and high probability protein targets of interaction for curcumin. Moreover, we have tried to correlate these targets with NAFLD using published literature on curcumin. Figure 1 provides an overview of the investigative strategy undertaken to perform this study.

## 2. Methods

Using the bioinformatics approach explores critical genes associated with curcumin and NAFLD in the online databases, then investigates their relations as targets of curcumin for NAFLD.

### 2.1. Curcumin and Target Search

We first searched interactions of curcumin in the STITCH database (http://stitch.embl.de/ (accessed on 3 October 2021)) to explore essential protein targets. STITCH is a platform for diagnosing interactions between chemicals and proteins. This database currently contains more than 9,640,000 proteins from 2031 organisms [33]. Here, we considered the high confidence cut-off (0.700) and limited the species only to Homo sapiens. We also illustrated the presence of proteins obtained in liver tissue based on the String plugin algorithm (1–5 score) in Cytoscape software (version 3.8.2).

### 2.2. Exploring Important NAFLD Genes in DISEASES and DisGeNET Databases

Next, we investigated the proteins on curated targets in two databases, the DisGeNET database (https://www.disgenet.org/ (accessed on 3 October 2021)) and the DISEASES database (diseases.jensenlab.org (accessed on 3 October 2021)), to find their association with NAFLD. DisGeNET is a database that contains a collection of genes associated with specific diseases. These data are integrated from a variety of sources, such as expert-curated repositories, the scientific literature and GWAS catalogs. DisGeNET currently covers more than 1700 genes and 24,000 diseases and traits [34]. For association genes with NAFLD, 1058 genes were registered in this database. Curated data contained seven primary resources: UNIPROT, ORPHANET, CTD, GENOMICS ENGLAND, CLINGEN, PSYGENET, and CGI. To achieve a curated dataset from DisGeNET, we used a plugin in the cystoscope to construct curated sources targets for NAFLD. This plugin contains different categories of existing sources in DisGeNET. The most important collection is curated data that are manually achieved. Therefore, we only used curated data by selecting CURATED from Select Source. DISEASES database is a weekly updated database that comprises diseases and gene relations from different resources, including manually curated literature, text mining, cancer mutation data, and genome-wide association research [35]. We extracted the targets from the available resources, including experiments and manually curated literature associated with NAFLD.

### 2.3. Venn Diagram to Obtain Important Curcumin Interaction Protein Targets in NAFLD

Before identification of common genes between two collections (curated genes-NAFLD and curcumin-STITCH), to eliminate the bias of different forms of gene names, all genes were first converted to UniProt by https://www.uniprot.org/uploadlists/ (accessed on 3 October 2021). For this purpose, we collected all genes and protein targets in different formats (gene symbols) and in the section “Retrieve/ID mapping” and converted to UniProtKB. We finally created a Venn diagram (http://bioinformatics.psb.ugent.be/webtools/Venn/ (accessed on 3 October 2021)) for these two sets of protein collections to find important targets of curcumin beyond the key recognized targets. A Venn diagram is a suitable method for identifying interesting overlaps between two or more sets.

### 2.4. Gene Ontology Pathway Enrichment Analyses for Target Proteins of Curcumin

Gene ontology (GO) enrichment is a popular procedure used to interpret genes and stratify them into three major categories: those that contribute to molecular function (MF), biological process (BP) or cellular component (CC) [36]. GO was analyzed for important targets obtained from the Venn diagram using the gene ontology resource with the web address: http://geneontology.org (accessed on 3 October 2021). Additionally, KEGG was analyzed using the Enrichr database with the web address: https://maayanlab.cloud/Enrichr/ (accessed on 3 October 2021). The KEGG pathway is a comprehensive database that maps pathways according to their metabolic inter-relationships [37]. In GO and KEGG analysis, the *p*-value < 0.01 was considered as the cut-off criterion. We also analyzed the enrichment pathways using the Wikipathway plugin in Cytoscape version 3.8.2. Cytoscape is a powerful software for integrating biomolecular interaction networks. It also has the practical capability to install multiple plugins to analyze high throughput data and different biological processes [38]. A *p*-value < 0.01 was considered as the cut-off criterion. In this plugin, we selected the Nonalcoholic fatty liver disease–Homo sapiens pathway and highlighted the curcumin protein interactions in this pathway based on their STITCH score. In addition, we estimated the specificity of obtained targets with NAFLD and investigated them in Tissue/Cells–gene associations (PanglaoDB Augmented 2021 and Mouse Gene Atlas) and the Disease–gene associations based EnrichR algorithm (Jensen DISEASES and DisGeNET). EnrichR is a free enrichment analysis website that possesses 180 184 annotated gene sets from 102 gene set libraries. It is a comprehensive online analyzer and engine searching machine to assemble biological knowledge [39]. PanglaoDB is a web server for probing single-cell RNA sequencing data, covering more than 1054 single-cell investigations from more than 4 million cells that derive from a broad range of tissues [40]. Mouse Gene Atlas is an online database resource for mouse research, providing integrated genomic, genetic, and biological information for promoting clinical studies in the field of human diseases [41].

## 3. Results

### 3.1. Protein Target Interaction with Curcumin in the STITCH Database

**Figure 2:**
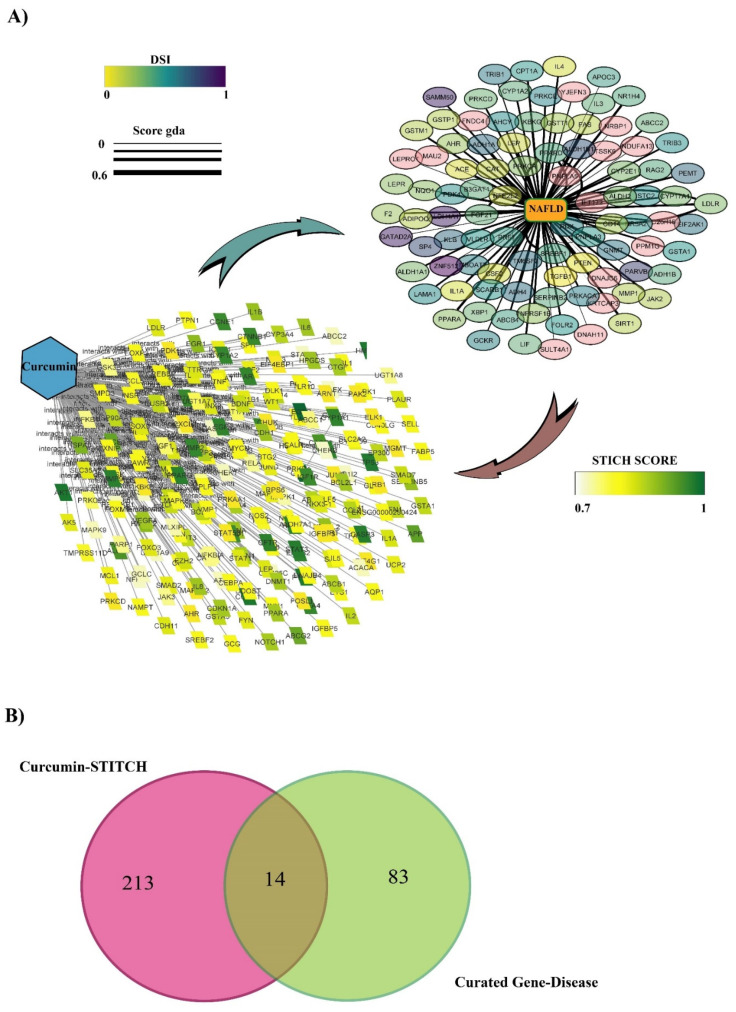
(**A**) Curated disease–gene database and curcumin–protein interaction visualized with Cytoscape software. For the curated disease-gene: Color intensity indicates the disease specificity index and the thickness of the edges indicates the genes–disease association score. For the curcumin–protein interaction, color intensity indicates STITCH score. (**B**) Venn diagram of the two datasets comprising the curated disease–gene database and curcumin-protein interaction.

Screening curcumin in the STITCH database with high confidence (0.7) identified 227 protein targets. The Drug–Protein interaction was visualized on Cytoscape (Figure 2A). STITCH scores are indicated by color intensity.

### 3.2. Discovering Curated NAFLD Genes

The curated data DisGeNet plugin on Cytoscape and DISEASES database identified 95 genes associated with NAFLD. All the data were visualized with Cytoscape software (Figure 2A). In this figure, the disease specificity index with genes (DSI g) is indicated by color intensity, and the genes–disease association score (gda score) is indicated with the thickness of the edges.

### 3.3. The Overlap of Curcumin Targets on the STITCH and Curated NAFLD Genes Were Visualized Using a Venn Diagram

A Venn diagram of the two created datasets revealed 14 candidates, CYP1A2, NFE2L2, PPARA, GSTA1, IL1A, LEP, LDLR, CSF2, GSTP1, PRKCE, TGFB1, ABCC2, AHR, and PRKCD (Figure 2B), that may be directly or indirectly affected by curcumin. Direct interaction refers to physical interaction, and indirect interaction refers to functional association. The scoring based on DisGeNET is shown in Table 1. It was noted that the highest relation with NAFLD belongs to TM6SF2 and PNPLA3, with gda scores of 6 and 5, respectively. However, due to the high confidence cut-off, seven candidates showed no relationship, and therefore we excluded them from our study.

### 3.4. GO and KEGG Enrichment Analyses of Protein Targets of Curcumin

GO analysis of the 14 identified protein targets demonstrated major involvement in response to oxygen-containing compounds, cellular response to lipid and stress, and regulation of lipid metabolic process under biological process (Table 2). This analysis additionally showed that these protein targets were chiefly involved in calcium-independent protein kinase C activity, signaling receptor activator and regulator activity, and transcription coregulator binding under the molecular function category. Furthermore, cellular components included endolysosome, extracellular space, and secretory granule lumen (Table 2).

In KEGG enrichment, we observed several biological pathways. The highest *p*-value pathways included chemical carcinogenesis, the AGE-RAGE signaling pathway in diabetic complications, fluid shear stress and atherosclerosis, non-alcoholic fatty liver disease and cytokine–cytokine receptor interaction (Table 3).

### 3.5. Specificity of 14 Obtained Protein Targets with Cells/Liver Tissue and Fatty Liver Disease in Databases

**Figure 3:**
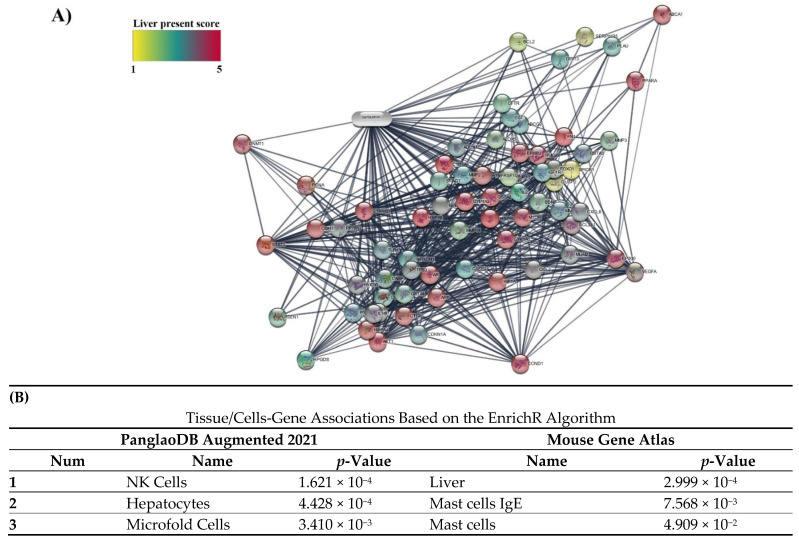
(**A**) Amount of expression of protein targets in interaction with curcumin in liver tissue. The intensity of color shows the amount of expression in the liver. (**B**) Association of 14 obtained protein targets in the interaction of curcumin with Tissue/Cells databases.

In Cytoscape software, the intensity of the color indicates the number of expressed genes that interacted with curcumin in liver tissue (1–5) (Figure 3). The score of expression of 14 obtained genes is reported in Table 1. Eight genes, including GSTA1, LDLR, PPARA, ABCC2, CYP1A2, PRKCD, GSTP1, and AHR, had high confidence scores (score 4). Additionally, the specificity of all 14 obtained targets in enrichment analysis for liver cells and tissues in two databases, PanglaoDB Augmented 2021 and Mouse Gene Atlas based on the EnrichR algorithm, was confirmed (Figure 3).

Moreover, the specificity of 14 obtained targets with NAFLD was confirmed based on the EnrichR algorithm in two databases, including Jensen DISEASES and DisGeNET. The result showed that NAFLD, and its advanced form NASH, were ranked in the top three in these databases (Table 4).

**Figure 4:**
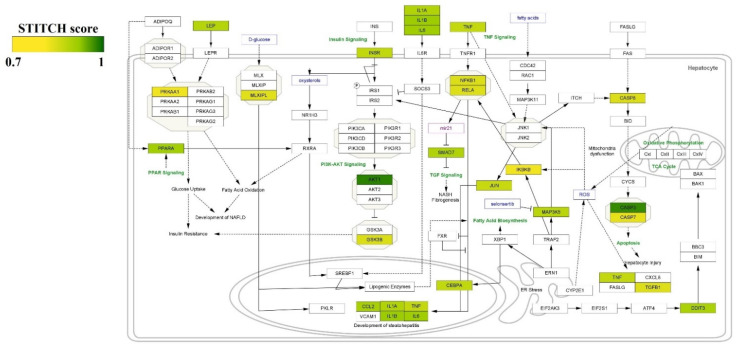
Visualizing protein interactions with curcumin in the NAFLD pathway with high confidence (0.7). The intensity of color illustrates the strength of interaction of curcumin with the targets.

Furthermore, we visualized all curcumin–protein targets with high confidence (0.7) in the NAFLD pathway in Wikipathway. The more intense color indicates greater interaction (based on the STITCH score) (Figure 4). The pathways are constructed using the Cytoscape with Wikipathway plugin (version 3.3.7).

## 4. Discussion

As a metabolic disorder, the importance of NAFLD has gained prominence in parallel with the accelerating prevalence of other metabolic syndromes [42]. In addition to causing metabolic dysfunction, NAFLD can progress and increase the risk of transforming to hepatocellular carcinoma if left untreated [43]. Identifying several important susceptibility genes that play a central role in the development and phenotype of NAFLD is a critical and initial step in order to determine suitable drugs for managing NAFLD. Curcumin has been shown to alleviate the pathological dysfunction of NAFLD and could exert these mitigating influences through effects on several important NAFLD-related genes [9,44,45,46]. Here, we investigated and analyzed possible protein targets of curcumin in databases and biological pathways, intending to understand the effects of curcumin in NAFLD in greater depth.

In the present study, we first searched significant predictions of protein interaction with high confidence for curcumin. Then, we probed protein interaction associated with fatty liver disease, determined its impact as an inhibitor, activator, binding, or transcription factor on these genes, and selected the most relevant targets. In doing so, we identified 14 significant targets: CYP1A2, NFE2L2, PPARA, GSTA1, IL1A, LEP, LDLR, CSF2, GSTP1, PRKCE, TGFB1, ABCC2, AHR, and PRKCD, and then evaluated their biological pathways. Most of the targets were involved in response to lipid and stress and lipid metabolism based on gene ontology. KEGG enrichment pathways showed chemical carcinogenesis, the AGE-RAGE signaling pathway in diabetic complications and NAFLD as the most common significant pathways. The targets we found among the authentic disease databases were more specific to fatty liver disease (steatosis and steatohepatitis) than other diseases in these databases. We also investigated the possible presence of these important protein targets in the liver. Those 14 protein targets that we identified were responsible for different stages of NAFLD, including steatosis, steatohepatitis, and fibrosis. We determined that curcumin could impact each of those stages through modulating essential protein targets.

PPARA (peroxisome proliferator-activator receptors-alpha) is a ligand-activated transcription factor, richly expressed in the liver and modulates various genes implicated in the catabolism of fatty acids [47]. Activation of PPARA promotes genes involved in oxidative phosphorylation and fatty acid beta-oxidation [48]. This activation has a protective effect in NAFLD with antioxidant, anti-inflammation, and lipid accumulation inhibition in the liver [49,50,51,52,53,54]. Numerous studies have been designed to activate PPARA for slowing the progression or curing NAFLD, and they demonstrate enhancement in metabolic activity and decrease of steatosis and fibrosis in NAFLD patients and models [48,55,56,57]. One study showed that curcumin activated PPARA and modulated several upstream signaling pathways (AMPK, PI3K/AKT/mTOR), which inhibit oxidative stress and increase autophagic flow in liver cells [58]. Another study reported that, in high-fat diet (HFD) mice, the level of expression of PPARA was reduced, whereas, after treatment with curcumin, the PPARA expression level was restored [59]. Of note, in our study (Table 1), PPARA had the third-highest score for interaction with curcumin and showed a strong relationship to NAFLD (gda score 0.4). This gene is extensively expressed in the liver (4.76), and curcumin was shown to activate PPARA (Table 1).

NFE2L2 (nuclear factor erythroid-derived 2-like 2) is a potent transcription factor in modulating xenobiotics and antioxidants in stress responses. However, oxidative stress causes the progression of NAFLD, and NFE2L2 could promote the reduction of reactive oxygen species (ROS) and amelioration of NAFLD [60]. In NFE2L2-knockout-mice, the ratio of reduced glutathione/oxidized glutathione (GSH/GSSG) decreased and steatohepatitis was severe [61]. Another study showed that NFE2L2 could be involved in cholesterologenic and lipogenic pathways, repressing fat accumulation and oxidative stress in HFD mice. In a study on hepatocyte-specific NFE2L2-upregulation mice with diet-inducing hepatic steatosis, thioredoxin-1, glutathione peroxidase-2, and Nqo1 were increased, and oxidative stress markers decreased. Further experiments also indicated that lipogenesis was inhibited and that the catabolism of lipid was increased in hepatocytes [62]. The protective role of overexpression of NFE2L2 against oxidative stress and reduced hepatic steatosis was demonstrated in mouse hepatic cells from a methionine–choline deficient (MCD) diet-induced NAFLD model [63]. In mice models with lipotoxicity, NFE2L2 was shown to mediate induction of SQTM1, enabling hepatoprotection under lipotoxic conditions [64]. NFE2L2 also was shown to regulate inflammation by repressing JNK (c-Jun *N*-terminal kinase) and the nuclear factor-kappa B (NF-κB) pathway in HFD diet mice.

Curcumin exerts its anti-diabetic, cardioprotective, hepatoprotective, neuroprotective, and antitumor effects via NFE2L2 signaling pathways [65]. Curcumin activates NFE2L2 signaling pathways in four ways: impacting mediators of NFE2L2, inhibition of KEAP1, impacting NFE2L2 expression, and reducing nuclear translocation of NFE2L2 [65]. In a high-fat and high-fructose diet (HFHFr) mouse model, NFE2L2 was downregulated, while curcumin administration could reverse the abnormal serum biochemical parameters of hepatic steatosis [66]. Another animal study using carbon tetrachloride (CCL4) induced liver damage showed that curcumin’s protective role in reducing inflammation and oxidative stress was mediated through NFE2L2/HO-1 pathways [67]. An in-vitro study showed that curcumin, through activation of NFE2L2, can promote lipocyte activation in stellate cells (HSCs) and repress hepatic fibrosis [68].

In our current study, curcumin demonstrated a robust interaction with NFE2L2 based upon the STITCH score, having the second-highest score after CYP1A2 (high confidence: 0.877), and its function was determined to be through activation of NFE2L2. Similarly, NFE2L2 had the highest score (0.4), together with leptin (LEP) and PPARA, in the gene-diseases association database (Table 1), indicating a significant role in the pathogenesis of NAFLD. For expression in liver tissue, however, it ranked ninth when compared with the other genes.

LEP was shown to be important with regards to NAFLD in our study (gda score: 0.4). LEP is a polypeptide hormone that interacts with its receptor, lepRb [69]. Several studies have investigated the role of leptin in NAFLD. Serum leptin levels are increased in both patients with NAFLD and in animal models of the disease and may be associated with the development of hepatocyte steatosis and its progression through OB-R (leptin receptor) activation of the PI3-K/Akt kinase pathway [70,71]. Moreover, leptin could be considered a surrogate biomarker for diagnosing patients with NASH/NAFLD.

Curcumin reduces the expression and secretion of leptin [72,73,74]. Askari et al. reported that curcumin delivered in a nanoparticle carrier caused a time and dose-dependent decrease in leptin levels [75]. Curcumin inhibits hepatic stellate cell (HSC) activation, while leptin induces HSCs which, in turn, cause progression of NASH to fibrosis. By decreasing phosphorylation of Ob-R, inducing PPAR-gamma, and reducing oxidative stress, curcumin inhibits Ob-R expression and blocks leptin signaling [72]. A recent study indicated that curcumin represses the methionine adenosyltransferase 2A (MAT2A) promoter that promotes leptin expression. MAT2A activity is linked with HSC activation and DNA methylation [76]. According to our STITCH results, curcumin had a significant interaction with LEP (0.82). Additionally, LEP had a robust relationship with NAFLD (gda: 0.4), but its expression was not specific to the liver (3.17).

Low-density apolipoprotein receptor (LDLR) is a mediator for cholesterol uptake in cells and is crucial for the clearance of cholesterol by the liver [77]. LDLR deficient rodents have been used to establish models of NAFLD [78,79]. In those models, elevations in hepatic neutral and hepatic pro-inflammatory oxylipins were observed [80]. An in-vitro study demonstrated that, by inducing fatty acid, the expression of LDLR was upregulated [81]. Curcumin was reported to induce LDLR expression on the cell surface and upregulate its activity in hepatic cells. Moreover, the uptake of LDL is significantly elevated in HepG2 cells following curcumin treatment [82,83], which might contribute to the clearance of cholesterol by the liver. Research on the CCl4 induced cirrhosis rat model also showed upregulation of LDLR expression with curcumin treatment [84]. With a STITCH score of 0.824 and a gda score of 0.37, our results show a strong relation of curcumin with LDLR and of LDLR with NAFLD diseases, respectively. As we show in Table 1, based upon its high score (4.41), the expression of LDLR is specific to the liver.

Cytochrome P450 isozyme 1A2 (CYP1A2) plays an important role in metabolizing drugs and carcinogens [85]. Research on high-fat and high-sucrose (HFHS) diet mice revealed an increase in the expression of CYP1A2 [86]. Additionally, the increased CYP profile, evidenced by increased CYP1A2, normalized after tetrahydro-curcumin administration in a study using the HFHS mouse model [87]. However, a study in humans reported that microsomal CYP1A2 decreased with NAFLD progression [88]. An in-vitro study using the palmitic acid (PA)-induced NAFLD model also indicated that expression of CYP1A2 was inhibited. Several studies have reported that curcumin downregulates the expression of CYP1A2 and, in this way, ameliorates the dysregulation of CYPs in various diseases [89,90,91,92].

In our study, CYP1A2 was identified as one of the most important NAFLD genes inhibited by curcumin (STITCH score: 0.948). According to our dataset, this gene is specifically present in the liver (high confidence: 4.78). However, due to conflicting results in studies about the increase and decrease of CYP1A2 in NAFLD, curcumin administration may benefit or exacerbate the disease, an issue that requires further investigation.

Several studies have shown that IL1α is increased in NASH/NAFLD patients [93,94,95] and is positively correlated with advanced stages of steatosis and steatohepatitis. IL1α was reported to be ~122% and ~300% higher in patients with steatosis and steatohepatitis than in controls [96]. Saberi-Karimian in 2020 reported that curcumin ameliorates inflammatory cytokines like IL1A in serum of patients with NAFLD, and curcumin exhibited anti-steatotic properties [29]. Curcumin was shown to modulate IL1A in a dose and time-dependent manner [97]. An investigation of the effect of curcumin on bone-marrow stromal cells showed that IL1-A was repressed [98]. Our study also reported that IL1A, with a gda score of 0.33, is associated with NAFLD, but its expression in the liver has a low score compared to the other protein target genes (2.78) in Table 1. However, the STITCH score of 0.829 indicates the possibility for curcumin to exhibit potent inhibition of IL1A.

Glutathione s-transferases (GSTs) are catalyzing enzymes that catalase hydrophobic and electrophilic compounds through glutathione reduction. GSTA1 is a member of the alpha class of GSTs and is principally expressed in the liver [99]. GSTA1 polymorphisms are associated with an increased risk of NAFLD [100]. In HepG2 induced lipotoxicity, the expression of GSTA1 was down-regulated [101]. In in-vivo studies, the expression of GSTA1 was decreased in fatty liver mouse models [102,103]. An in-silico docking study revealed that curcumin exhibited a potent affinity to GST isoforms such as GSTA1 [104], and that curcumin could modulate the expression of GSTA1 [105,106]. Here, we showed that curcumin, with a high confidence score of 0.846, influences GSTA1 in both activation and inhibition. Furthermore, with a gda score of 0.31, GSTA1 is a critical protein target in NAFLD and was also shown here to be the most specific protein target in the liver, based on a score of 4.90, in comparison with other identified protein targets.

Glutathione S transferase Pi 1 (GSTP1) is another GST enzyme that plays a vital role in antioxidant defense through detoxifying foreign substances and inactivating byproducts of oxidative stress [107,108]. Research in 2009 by Hori et al. demonstrated for the first time that GSTs could be involved in the progression of NAFLD [108]. Proteomic analysis of GST proteins showed that pi1, mu1, and selenium binding protein-2 were reduced in diet-induced hepatic steatosis [109]. Moreover, several studies have reported that polymorphisms of GSTP1 are common in patients with NAFLD [110,111]. Various studies have investigated the effects of curcumin on GSTP1. An in-vitro investigation by Nishinaka et al. showed that antioxidant response element (ARE) is the primary region where curcumin induces transactivation of the GSTP1 gene [112]. Other research indicates that curcumin might induce apoptosis due to its inhibition of GSTP1 expression at the transcription level [113]. A 2015 study reported that mRNA and protein levels of GSTP1 in curcumin-treated groups were significantly lower than in control groups in human colon carcinoma cells [114]. In our data, curcumin was shown to be a transcriptional regulator of GSTP1 with high confidence (0.794). GSTP1 was present in the liver with a high confidence STITCH-based tissue score (4.64) and a close relationship with NAFLD (gda score: 0.33).

TGF-β is a growth factor and cytokine with profibrotic properties and is the most abundant isoform in the liver. TGF-βis also a key inducer of reactive oxygen species (ROS) [115]. A study in humans reported that profibrotic/scar deposition genes such as TGFB1 are elevated in steatohepatitis, which is purported to be due to the dynamic state of tissue remodeling [116]. In both mouse and human liver, TGFB1 as a pro-fibrogenic marker associated with NAFLD was upregulated [117]. A recent study reported that curcumin, an inhibitor of TGF-beta, also inhibits the transition of hepatocytes to myofibroblasts, a critical step in the progression of liver fibrosis [58]. A 2017 study reported that curcumin inhibited TGFβ1-induced Smad3 phosphorylation by conjunctival fibroblasts [118]. Further research demonstrated that curcumin decreases TGFB1, thereby delivering beneficial effects in resolving fibrosis [119]. High-dose curcumin was reported to significantly decrease the serum level of TGFB1 [120]. As shown here in Table 1, TGFB1 was 1 of the 14 important genes that interacted with curcumin and has a strong association with NAFLD. However, its distribution is not specific to the liver.

Colony-stimulating factor 2 (CSF2) has a role in binding to extracellular proteoglycans and modulating biological function [121]. CSF2 is produced by various cells, including fibroblasts, endothelial cells, macrophages, and most inflammatory cells, and, accordingly, as shown here in Table 1, CSF2 is not specific for hepatocytes. CSF2 is an important factor in the progression of liver fibrosis, and anti-CSF2 has been shown to ameliorate liver fibrosis [122]. CSF2 is increased in NASH patients and animal models of NASH [93,123,124,125]. Curcumin was reported to repress CSF2 in a concentration-dependent manner in allergic diseases [126]. Administration of curcumin to cancer patients resulted in a decrease in CSF2 [127]. In line with previous studies, our bioinformatics analysis shows that CSF2 has a strong relationship with NAFLD (gda score 0.3) and is robustly inhibited by curcumin (high confidence-based STITCH score: 0.816).

PRKCE is a member of the protein kinase C family, and has a C2 conserved domain (non-Ca2+ binding) and C1A and C1B domains for binding diacylglycerol (DAG) [128]. Several studies have reported that the accumulation of DAG causes activation of PRKCE, which leads to repression of insulin receptor kinase. This process induces hepatic insulin resistance, the most prevalent complication in NAFLD patients [129,130,131]. Curcumin binds to a specific domain attached to PRKCE and inhibits its activity [132]. Wang et al. in 2016 demonstrated that curcumin represses the accumulation of DAG and PRKCE translocation in the liver, which improves insulin function and inhibition of hepatic gluconeogenesis [133]. In our analysis, PRKCE was one of the protein targets discovered from 95 curated proteins in databases associated with NAFLD. Moreover, its binding activity with curcumin showed high confidence: 0.763. However, its distribution was not specific to the liver, showing less specificity than the other 14 protein targets.

ABCC2 is mainly expressed in significant physiological barriers such as the canalicular membrane in liver cells. It contains multiple binding sites and shows complex transport kinetics [134]. ABCC2 variants have been found to have a role in the susceptibility to and progression of NAFLD [135]. In patients with NASH, the expression of multiple efflux transporters was elevated, and cellular localization of ABCC2 is altered, which affects the elimination of drugs. According to our data, ABCC2, with a gda score of 0.32, has a relationship with NAFLD, and its presence in the liver was second only to GSTA1. An in-vitro study on hepatocellular carcinoma cell lines showed that ABCC2 has a critical role in hypoxia-induced resistance by curcumin [136]. More recent research revealed that curcumin could improve drug resistance, reversing the increased drug resistance of T24 cells treated with gemcitabine [137]. Curcumin may exert its effect by modulating the expression of ABCC2 [138]. However, as we show in Table 1, its STITCH score showed a low connection compared with most of the protein obtained (high confidence: 0.727).

Aryl hydrocarbon receptor (AHR) is a ligand-activated transcription factor with multifunctional activity and may benefit or adversely affect NAFLD [139]. Activation of AHR might positively improve NAFLD and may also have an anti-inflammatory effect in the disease [140]. AHR is highly expressed in the liver (4.40) and, with a gda score of 0.33 is highly associated with NAFLD. As a chemopreventative agent in cancer, curcumin has been shown to decrease AHR levels [141]. Curcumin has been shown to repress 3-methylcholanthrene binding (an AHR agonist) to the receptor; curcumin can bind to the AHR as a ligand and inhibit its transformation via phosphorylation, possibly acting through PKC [142]. However, several contradictory reports indicate that curcumin might decrease or increase AHR. Our data showed that curcumin was less associated with AHR than other targets with a STITCH score of 0.700 (Table 1).

Protein kinase c delta (PRKCD) is a member of the lipid-activated PKC family, which can be activated with DAG but not Ca^2+^ [143]. PRKCD has been reported to have a role in the progression of NAFLD [144]. PRKCD modulates SERCA (Sarco/endoplasmic reticulum calcium ATPase) activity in the human liver cell line (LO-2) induced with palmitic acid, an in-vitro model for steatosis [143]. SERCA is an important component in Ca^2+^ homeostasis [145]. Triglyceride (TG) accumulation was associated with dysregulation of PKCD and ER stress [143]. Deletion of PLDD has been shown to cause a reduction in lipogenesis by inducing important transcription factors in the liver in a HFD mouse model [146,147]. Deletion of PRKCD caused a decrease in TG accumulation and lipid droplet accumulation [143]. Various studies indicate that curcumin can stimulate PKCD phosphorylation, subsequently inducing downstream signaling pathways [148,149,150]. In our analysis, PRKCD was 1 of the 14 obtained protein targets (gda score: 0.31) with low association (STITCH score: 0.7) with curcumin in comparison with the other targets.

## 5. Conclusions

In summary, we surveyed the important NAFLD genes and their potential in targeting curcumin, together with their biological pathways. In this study, we identified 14 genes in NAFLD that are likely to be the target of curcumin and observed that curcumin induces or inhibits them. According to our results, this activity of curcumin was in line with improving NAFLD based on literature. We believe that the ongoing clinical trials investigating the effect of curcumin on fatty liver could yield positive results in the future, enhancing the therapeutic status of curcumin in this metabolic disorder.

## Figures and Tables

**Table 1:** The relationship of genes associated with NAFLD that are targets of curcumin (www.disgenet.org (accessed on 3 October 2021)).

Gene Symbol	UniProt	Gene_Full_Name	Protein Class	DSI g	Score Gda	STITCH			
						Score	Source	Action Type	Liver Tissue
**CYP1A2**	P05177	cytochrome P450 family 1 subfamily A member 2	Enzyme	0.49	0.30	0.94	Curated databases	Inhibition	4.78
**NFE2L2**	Q16236	nuclear factor, erythroid 2 like 2	Enzyme	0.35	0.40	0.87	Textmining	Activation	3.65
**PPARA**	Q07869	peroxisome proliferator-activated receptor alpha	Nuclear receptor	0.43	0.40	0.86	Curated databases	Activation	4.75
**GSTA1**	P08263	glutathione S-transferase alpha 1	Enzymes, Plasma proteins	0.56	0.31	0.84	Textmining	Activation/ Inhibition	4.90
**IL1A**	P01583	interleukin 1 alpha	Plasma proteins, Transporters	0.33	0.33	0.82	Textmining	Inhibition	2.77
**LEP**	P41159	leptin	Plasma protein	0.34	0.40	0.82	Textmining	Activation/Inhibition	3.17
**LDLR**	P01130	low density lipoprotein receptor	Plasma protein	0.44	0.37	0.82	Textmining	Activation/Inhibition, Transcriptional regulator	4.40
**CSF2**	P04141	colony-stimulating factor 2	Signaling	0.33	0.30	0.81	Textmining	Inhibition, Transcriptional regulator	2.60
**GSTP1**	P09211	glutathione S-transferase pi 1	Enzyme	0.38	0.33	0.79	Textmining	Activation	4.64
**PRKCE**	Q02156	protein kinase C epsilon	Kinase	0.59	0.31	0.76	Curated databases	Binding	2.34
**TGFB1**	P01137	transforming growth factor-beta 1	Signaling	0.28	0.34	0.73	Curated databases	Inhibition, Transcriptional regulator	3.01
**ABCC2**	Q92887	ATP binding cassette subfamily C member 2	Transporter	0.48	0.32	0.72	Curated databases	Inhibition	4.78
**AHR**	P35869	aryl hydrocarbon receptor	Transcription factor	0.41	0.33	0.70	Textmining	Unspecific	4.39
**PRKCD**	Q05655	protein kinase C delta	Kinase	0.48	0.31	0.70	Curated databases	Unspecific	4.49

DSI g: Disease specificity index for the gene, Score gda: Genes-disease associate score.

**Table 2:** Gene ontology enrichment analysis via Enrichr for the 14 identified genes with the best score interaction with curcumin.

**Biological Process (GO)**				
**Accession**	**Pathway Description**	**Gene Count**	***p*-Value**	**FDR**
**GO: 1901700**	response to oxygen-containing compound	13	3.93 × 10^−14^	6.17 × 10^−10^
**GO: 0071396**	cellular response to lipid	10	5.70 × 10^−14^	4.48 × 10^−10^
**GO: 0033993**	response to lipid	11	1.57 × 10^−13^	8.25 × 10^−10^
**GO:0006950**	response to stress	12	2.19 × 10^−0.08^	1.07 × 10^−0.05^
**GO:0019216**	regulation of lipid metabolic process	6	4.75 × 10^−0.08^	2.07 × 10^−0.05^
**Molecular Function (GO)**				
	**Pathway Description**	**Gene Count**	***p*-Value**	**FDR**
**GO:0004699**	calcium-independent protein kinase C activity	2	2.57 × 10^−0.06^	1.25 × 10^−0.02^
**GO:0030546**	signaling receptor activator activity	5	1.43 × 10^−0.05^	3.49 × 10^−0.02^
**GO: 0001221**	transcription coregulator binding	3	2.22 × 10^−0.05^	3.61 × 10^−0.02^
**GO: 0030545**	signaling receptor regulator activity	5	2.24 × 10^−0.05^	2.73 × 10^−0.02^
**Cellular Component (GO)**				
	**Pathway Description**	**Gene Count**	***p*-Value**	**FDR**
**GO: 0036019**	endolysosome	2	1.72 × 10^−0.04^	3.42 × 10^−0.01^
**GO: 0005615**	extracellular space	8	1.11 × 10^−0.06^	1.10 × 10^−0.03^
**GO: 0034774**	secretory granule lumen	3	1.23 × 10^−0.03^	8.16 × 10^−0.01^

FDR (false discovery rate) is an accurate statistical method permitting multiple comparisons while conserving a low false-positivity rate.

**Table 3:** KEGG pathways for 14 critical genes interact with curcumin.

	KEGG			
NUM	Pathway Name	Genes	Gene Count	*p*-Value
**1**	Chemical carcinogenesis	GSTP1, CYP1A2, GSTA1, AHR, PPARA	5	4.285 × 10^−7^
**2**	AGE-RAGE signaling pathway in diabetic complications	IL1A, TGFB1, PRKCE, PRKCD	4	5.667 × 10^−7^
**3**	Fluid shear stress and atherosclerosis	NFE2L2, IL1A, GSTP1, GSTA1	4	2.119 × 10^−6^
**4**	Non-alcoholic fatty liver disease	IL1A, PPARA, TGFB1, LEP	3	3.270 × 10^−6^
**5**	Cytokine-cytokine receptor interaction	IL1A, CSF2, TGFB1, LEP	4	5.667 × 10^−7^

**Table 4:** Association of protein targets obtained in the interaction of curcumin with the indicated disease in two databases.

Disease–Gene Associations Based on EnrichR Algorithm				
Jensen DISEASES			DisGeNET	
NUM	Name	*p*-Value	Name	*p*-Value
**1**	Arthritis	6.740 × 10^−6^	Non-alcoholic Fatty Liver Disease	3.411 × 10^−20^
**2**	Coronary artery disease	9.900 × 10^−6^	Nonalcoholic Steatohepatitis	1.206 × 10^−17^
**3**	Fatty liver disease	2.607 × 10^−5^	Hypertensive disease	2.505 × 10^−17^

## Data Availability

All data pertaining to this study will be made available upon reasonable request to the corresponding author.
